# Autorização para uso *off-label* pode não ser benéfica para o Sistema Único de Saúde

**DOI:** 10.1590/0102-311XPT085423

**Published:** 2023-06-26

**Authors:** Rosângela Caetano, Luciane Cruz Lopes, Gustavo Mendes Lima Santos, Claudia Garcia Serpa Osorio-de-Castro

**Affiliations:** 1 Instituto de Medicina Social Hesio Cordeiro, Universidade do Estado do Rio de Janeiro, Rio de Janeiro, Brasil.; 2 Programa de Pós-graduação em Ciências Farmacêuticas, Universidade de Sorocaba, Sorocaba, Brasil.; 3 Agência Nacional de Vigilância Sanitária, Brasília, Brasil.; 4 Escola Nacional de Saúde Pública Sergio Arouca, Fundação Oswaldo Cruz, Rio de Janeiro, Brasil.

O debate é fundamental e agradecemos o interesse e a contribuição de tantos colegas em discutir nosso trabalho publicado em CSP. Nosso artigo ^1^ se debruçou sobre as novas normativas quanto ao uso *off-label*, que chegaram como “decreto”, e suas potenciais repercussões, diante do papel institucional da Agência Nacional de Vigilância Sanitária (Anvisa) e da relevância do registro para a saúde pública. Nesse sentido, medicamentos são registrados e a indicação de registro é a indicação autorizada pelo país, com discricionariedade da Anvisa.

Verificamos que o conteúdo da Carta às Editoras ^2^, de fato, não aborda o assunto central do nosso artigo: a nova normativa e suas potenciais consequências. Ademais, discordamos quanto à chamada para exame do contexto. Todo nosso artigo é sobre o contexto. Qual é o “contexto” a que os colegas se referem? Particularizar algumas situações da Comissão Nacional de Incorporação de Tecnologias no Sistema Único de Saúde (Conitec) não significa oferecer contexto da incorporação no país, ao contrário, apenas individualiza recomendações que podem ter sido boas em alguns casos, porém bastante controversas em outros, como nos medicamentos para atrofia muscular espinhal (AME). Naqueles casos, algumas recomendações foram, ao contrário do que os colegas afirmam, baseadas em outra perspectiva, pois as evidências que as embasaram não se modificaram ao longo do tempo, enquanto as recomendações, sim ^3^. Ou se basearam em estudos comparativos desenvolvidos pela indústria, já que naquele momento não havia outros ^4^.

O uso *off-label* é um instrumento que pode - e é - usado pela indústria para cobrir seus interesses, de formas variadas ^5^. Caso o medicamento seja recentemente incorporado, uma forma efetiva de expansão de mercado é por meio do uso *off-label*. Por outro lado, em alguns nichos terapêuticos de maior dinamismo tecnológico, com inúmeras opções terapêuticas, esse uso já não se torna tão interessante, pois haverá maiores ganhos com a multiplicidade de incorporações. Assim, o contexto é muito importante - é ele que determina qual a faceta a ser mobilizada pela indústria para influenciar ou não o uso *off-label*.

Outrossim, o uso *off-label* pode ser útil em situações que gerem maior acesso e possibilidades de tratamento adequado em casos muito específicos. Há vários exemplos na prática clínica, sendo o mais contundente o uso pediátrico. No entanto, institucionalizar, *a priori*, o uso *off-label* carreia riscos que não podem ser minimizados ^6^. Nesse sentido, há preocupação da agência com os planos de farmacovigilância e gerenciamento de riscos, justamente nas populações mais vulneráveis, crianças, idosos, gestantes, em que esse uso é mais prevalente.

A qualidade das evidências para uso *off-label* é muitas vezes frágil, sobretudo porque os medicamentos não foram expostos à testagem e avaliação que fundamentam o registro. O uso *off-label* pode “liberar” a indústria do desenvolvimento de novos ensaios para a nova indicação pretendida, auferindo economia de recursos e muitos lucros. No emprego *off-label*, as consequências do “não uso” não são apresentadas. A ausência de aprovação pelo órgão regulador implica falta de faixas de doses seguras e de informações adequadas sobre contraindicações. Resultados de eficácia negativos ou não significativos não são publicados. As evidências de eventos adversos são prejudicadas pela pequena abrangência do uso na indicação não registrada, e não há estímulo para estudos Fase IV ^6^^,^^7^^,^^8^.

Ainda, a responsabilidade pelo uso *off-label* é do profissional prescritor, contrastando com a responsabilidade compartilhada do uso indicado, que tem na agência reguladora seu garantidor. Os fabricantes têm pouca responsabilidade legal em relação à prescrição, pois só podem ser responsabilizados por problemas que surgem quando seu medicamento é utilizado de acordo com as indicações aprovadas. O uso *off-label* promove crescimento do mercado sem aumento de responsabilidades com as consequências do uso ^9^.

O contexto da provisão, consequência necessária da incorporação e parametrizada por prazos, é de exame essencial. É fato que nem sempre os prazos de disponibilização no Sistema Único de Saúde (SUS) são cumpridos ^10^^,^^11^, mesmo para usos *on-label*, em que a necessidade pode ser estimada com maior certeza, pela previsibilidade. A atual variabilidade de abordagens terapêuticas no âmbito do SUS já apresenta iniquidade importante e documentada ^12^^,^^13^. Quais seriam os contextos da provisão e do tratamento em cenário de incorporações diversas e uso *off-label*? E não estamos adentrando o contexto da alocação de recursos, em que as prioridades deveriam estar em pauta, em situação de recursos limitados e judicialização galopante.

É de competência exclusiva da Anvisa a decisão sobre como um medicamento deve ou não ser registrado, com processos definidos e equipe com *expertise*. Imaginar que outras instâncias estariam mais qualificadas para realizar o trabalho da agência seria não apenas desconsiderar sua competência, mas abrir espaço para que outros também desqualifiquem a competência da instância responsável pela avaliação de tecnologias hoje empreendida para o SUS.

Sobre a nova estrutura da Conitec, mantemos a impressão de que atomiza a responsabilidade sobre as recomendações e pode fragmentar a integralidade das decisões, mas estamos abertos a novos desdobramentos positivos, no decorrer dos seus trabalhos no novo formato. Ressalta-se, ainda, que nosso trabalho não realizou uma análise da qualidade dos processos da Conitec. Sentimos que os autores da carta deveriam dedicar-se a escrever mais sobre essa relevante questão, o que seria de grande utilidade para a academia e para o fortalecimento da Conitec.

Pressões sociais e políticas fazem parte dos cenários de avaliação e de incorporação em todo o mundo. No Brasil, temos alguns bons exemplos, e outros nem tanto, como os casos da fosfoetanolamina, da cloroquina e de outros medicamentos reposicionados para COVID-19, em uso *off-label*, reforçando a necessidade de estudos bem conduzidos, que possam garantir, no mínimo, eficácia e segurança.

Concordamos com os colegas que a participação dos segmentos sociais na Conitec é ainda insuficiente, concentrando-se nos grupos com interesse explícito nas incorporações, por variados motivos, e deixando de fora todos os que não têm os mesmos interesses - os quais representam, muitas vezes, a parcela mais significativa - e o SUS universal ^14^. Aprovamos iniciativas que visem influenciar a revisão dessas participações, de modo a garantir uma representação plural e abrangente dos interesses sociais de todos.

Esperamos ter gerado reflexões sobre as eventuais consequências da legislação citada. O conceito *off-label* abarca múltiplas vertentes e exige consciencialização e responsabilidade constantes no seu exercício, tendo a Anvisa competência exclusiva por esse tipo de uso no Brasil.

## References

[1] Caetano R, Lopes LC, Santos GML, Osorio-de-Castro CGS (2023). Incorporação e uso de medicamentos no Sistema Único de Saúde
mudanças e riscos com os novos atos normativos do Ministério da
Saúde. Cad Saúde Pública.

[2] Santos MS, Costa MGS, Tura BR, Torres P, Martins SJ, Toscas FS (2023). Autorização para uso off-label pode ser benéfica para o Sistema
Único de Saúde?. Cad Saúde Pública.

[3] Caetano R, Hauegen RC, Osorio-de-Castro CGS (2019). A incorporação do nusinersena no Sistema Único de Saúde uma
reflexão crítica sobre a institucionalização da avaliação de tecnologias em
saúde no Brasil. Cad Saúde Pública.

[4] Ribero VA, Daigl M, Martí Y, Gorni K, Evans R, Scott DA (2022). How does risdiplam compare with other treatments for types 1-3
spinal muscular atrophy a systematic literature review and indirect
treatment comparison. J Comp Eff Res.

[5] Stafford RS (2008). Regulating off-label drug use rethinking the role of the
FDA. N Engl J Med.

[6] Eguale T, Buckeridge DL, Verma A, Winslade NE, Benedetti A, Hanley JA (2016). Association of off-label drug use and adverse drug events in an
adult population. JAMA Intern Med.

[7] van der Zanden TM.Smeets NJL.de Hoop-Sommen M.Schwerzel MFT.Huang
HJ.Barten LJC (2022). Off-label, but on-evidence A review of the level of evidence for
pediatric pharmacotherapy. Clin Pharmacol Ther.

[8] Wong J, Motulsky A, Abrahamowicz M, Eguale T, Buckeridge DL, Tamblyn R (2017). Off-label indications for antidepressants in primary care
descriptive study of prescriptions from an indication based electronic
prescribing system. BMJ.

[9] Verbaanderd C, Rooman I, Meheus L, Huys I (2020). On-label or off-label Overcoming regulatory and financial
barriers to bring repurposed medicines to cancer patients. Front Pharmacol.

[10] Capucho HC, Brito A, Maiolino A, Kaliks RA, Pinto RP (2022). Incorporação de medicamentos no SUS comparação entre oncologia e
componente especializado da assistência farmacêutica. Ciênc Saúde Colet.

[11] (2023). Demora na incorporação de medicamentos ao SUS penaliza pacientes
com doenças raras.. JOTA.

[12] Kaliks RA, Matos TF, Silva VA, Barros LHC (2019). Diferenças no tratamento sistêmico do câncer no Brasil meu SUS é
diferente do teu SUS. Braz J Oncol.

[13] Silva MJS, Osorio-de-Castro CGS (2019). Organização e práticas da assistência farmacêutica em oncologia
no âmbito do Sistema Único de Saúde. Interface (Botucatu).

[14] Lopes ACF, Novaes HMD, Soárez PC (2020). Patient and public involvement in health technology
decision-making processes in Brazil. Rev Saúde Pública.

